# Current Knowledge and Novel Therapeutic Approaches Based on Pharmacokinetics and Pharmacodynamics in Stress-Induced Pathology

**DOI:** 10.3390/medicina58070839

**Published:** 2022-06-22

**Authors:** Cornelia Amalinei

**Affiliations:** Department of Morphofunctional Sciences I, “Grigore T. Popa” University of Medicine and Pharmacy, 700115 Iasi, Romania; cornelia.amalinei@umfiasi.ro

There have been numerous progresses recently made in the knowledge of different types of stress involvement in human pathology, in an effort to counteract or to prevent their etiopathogenic pathways or to find novel therapeutic approaches. Given the numerous types of stress factors or stressors, a brief classification of different kinds includes environmental (thermal stress, UVR or viral infections), metabolic (high body-mass index), and drug-related (alcohol, nicotine, anaesthetic drugs or opioids), in association with oxidative stress.

Many epidemiological studies have demonstrated an increased risk of death related to exposure to extreme temperatures, in correlation to temperature threshold, humidity, and the lagged effect of temperature on mortality [1,2]. Current knowledge indicates that monocytes are responding to cold by a delayed and prolonged secretion of the proinflammatory cytokines, such as interleukins (IL-1β and IL-6) and tumour necrosis factor α (TNF-α), leading to capillary leakage, tissue damage, and ultimately shock, while catecholamines and cortisol are important players in the thermoregulatory defence mechanisms in cold exposure [3]. On the other hand, therapeutic hypothermia may be used as a standard care, mainly for term and near-term neonates with neonatal encephalopathy [4], in traumatic brain injury, ischemic stroke, cardiac arrest, or during various types of surgery. At the other extreme, hyperthermia may occur in high ambient temperatures, mainly affecting people with various constitutional or medical conditions, which increase their susceptibility to fail in heat-liberation mechanisms or lead to drug-related hypohidrosis associated with the use of alcohol, antihistamines, antiparkinsonians, anticholinergics, antipsychotics, antidepressants, amphetamines, cocaine, with heat exhaustion and stroke being life-threatening conditions for these individuals [2].

Ultraviolet radiation (UVR) is an important environmental stressor, considering its ability to induce oxidative DNA damage, by wavelength-dependent manner photoproducts, such as cyclobutane pyrimidine dimers (CPD) and 6-4-pyrimidone photoproducts (6-4PP), along with UV-induced reactive oxygen species (ROS), leading to melanoma [5]. Although melanocytes are protecting against UV-induced DNA damage, variable factors, either genetic, such as melanocortin-1 receptor (MC1R) inherited variants or mutations of tumour suppressor genes, or epigenetic histone modifications in melanocytes result in an increased melanoma susceptibility [6]. UVR is also responsible for the immunity suppression, by antigen presentation inhibition, immunosuppressive cytokines, and immune cells apoptosis. Consequently, novel immunotherapies are nowadays available, such as combination therapy, associating c-Kit inhibitors with therapies targeting its downstream pathways or anti-BRAF, anti-MAPK proteins, along with anti-PD-1 and CTLA-4, with different immune checkpoint proteins being currently evaluated as possible markers for novel therapy strategies, along with innovative therapies, such as those of microbiota transfer [6].

Numerous evidences, certified by molecular techniques and culture methods, confirm the origin of various chronic syndromes from viral infectious agents—for instance, the association between Epstein-Barr virus and Burkitt lymphoma, Human papillomavirus and cervical cancer, hepatitis B virus (HBV) infection and chronic liver disease as well as hepatocellular carcinoma, hepatitis C virus and mixed cryoglobulinemia, KSHV (human herpesvirus 8) and Kaposi sarcoma, poliovirus as well as West Nile virus and paralysis, Human T-lymphotrophic virus type 1 and tropical spastic paraparesis as well as chronic arthropathy [7], and, more recently demonstrated, SARS-CoV-2 and chronic fatigue syndrome (myalgic encephalomyelitis) [8]. The mechanisms involved in their pathogeny are related to direct effects, immune response, or persistent, progressive tissue pathology. Although some of these infections are preventable due to vaccination, such as HPV and HBV, others still need further research regarding their pathogenesis, by the combined use of histopathology, genomics, proteomics, microarrays, mass spectrometry, and nanotechnology, in order to certify the mechanisms and effects of in-viral aggression, as well as to identify novel modalities to target different molecules or pathways by personalized therapies.

Increased white adipose tissue (WAT) accumulation, especially of visceral type, is correlated with metabolic stress or metabolic diseases. WAT hypertrophy or hyperplasia have been analysed in adults with body-mass index (BMI) ≥30 or percentage of body fat ≥35% in women or ≥25% in men, according to World Health Organization (WHO) diagnosis criteria [9]. Currently, obesity is considered as a chronic mild inflammation, associated with macrophage infiltration, and adipocyte hypoxia, leading to disturbances of hormones and cytokines that are regulators of metabolism, exacerbating the obesity pathogenesis [9,10]. Moreover, a disturbed stress response, with hyperstimulation of the hypothalamus-pituitary-adrenal axis, greater production of corticotropin-releasing factor (CRF), as stress response initiator and adrenocorticotropic hormone (ACTH) release, as promoter of glucocorticoids secretion, has been demonstrated in obese people. Cortisol level increase in obesity may also result from the increase of WAT mass, with 11β-hydroxysteroid dehydrogenase type 1 (11βHSD1) enzyme that transforms the inactive cortisone into cortisol, which promotes hepatic gluconeogenesis. Additionally, insulin secretion is activated, along with lipoprotein lipase (LPL) stimulated activity, to further enhance the visceral WAT accumulation. Not surprisingly, a correlation between urinary cortisol metabolite (5α- and 5β-tetrahydrocortisol) excretion and BMI has been also observed in obese patients. Additionally, leptin resistance in obese people leads to a reduced lipolysis, insulin sensitivity, and fatty acid oxidation, and it is also associated with enhanced insulin and hepatic glucose production. Due to increased circulating insulin and glucose, added to reduced insulin sensitivity, hyperinsulinemia and hyperglycaemia tend to develop, in association with hyperlipidaemia, due to decreased fatty acid oxidation, and this process completes the spectrum of obesity metabolic disturbances. Furthermore, decreased adiponectin level is also correlated with dyslipidaemia in obese people. The increased metabolic demand may be initially compensated by an enhanced β-cell mass, which is able to increase insulin secretion, but insulin resistance will further increase to overt diabetes due to progressive dysfunction and apoptosis of β-cells [9].

Environmental factors, such as nutritional stressors or pollutants, including exposure to pesticides, can be also associated with the onset of obesity by epigenetic modifications, increasing the risk of diabetes development. Interestingly, chronic elevation of glucocorticoids induced by stress leads to stimulation of adipocytes leptin production and pancreatic insulin, followed by a reduced brain sensitivity for both of them, resulting in leptin and insulin resistance and, consequently, appetite suppression loss [9]. Additionally, many heritable features correlated to lipid metabolism, appetite regulation, and inflammation have been demonstrated in obesity. It is considered that epigenetic modifications are preceded by physiological alterations, while stressors may promote obesity pathogenesis by adipose tissue epigenetic modifications or by stress response, and feeding behaviour may promote hypothalamic regulation [9].

The increased glucose levels in diabetes results in an increased production of reactive oxygen species (ROS), associated with impairment of normal body antioxidative defence mechanisms, resulting in oxidative stress or tissue damage through different signalling pathways, promoting diabetic complications. ROS-induced oxidative stress results in protein modifications, by carbonylation, nitrosylation, glutathionylation, and glycation, with their accumulation, mediating the advance of diabetes pathogenesis, and this process contributes to hyperglycaemia-induced macro- and microvascular complications [11]. Moreover, evidences of recent studies indicate that oxidative stress is not only playing a critical role in diabetes development and its cardiovascular complications but is also involved in other metabolic and neurodegenerative disorders, such as renal disease, Alzheimer’s disease (AD) [12], ageing, and cancer, which may be possibly overcome by exogenous sources of antioxidants [11]. The deep insight into the stress-induced disturbances of adipose tissue mechanisms may lead to the development of novel therapeutic approaches in the management of stress-induced metabolic diseases and obesity.

Recent progresses have been registered in pharmacokinetics (PK), related to absorption, metabolism, distribution, and elimination of medications, pharmacodynamics (PD), related to the response of stimulation of the drug target receptor, or ADME (absorption measured by bioavailability- F, distribution, measured by volume of biodistribution- Vd, metabolism, and elimination, measured by elimination rate constant- k_e_ or drug clearance- CL), as basic pharmacokinetic parameters. In correlation with the drug structure, different families of enzymes perform its metabolism, such as cytochrome P450s (CYPs), epoxide hydrolases (EH), flavin-containing monoxygenases (FMO), N-acetyltransferases (NAT), UDP-glucuronosyltransferases (UGT), glutathione S-transferases (GST), and sulfotransferases (SULT) [13], resulting in modification of the drug’s pharmacokinetics, pharmacodynamics, and potential toxicity profiles. These enzymatic reactions may frequently lead to toxic metabolites correlated to drug-related stress, oxidative stress, carcinogenicity, cell death, or teratogenesis [14].

Alternatively, numerous pro-drugs have been obtained by metabolic activation, characterized by less generalized toxicity and high levels of biologically active molecules in the target tissues. This metabolic activation is catalysed by cytochromes, including levodopa, diazepam, cyclophosphamide, talampicillin, ftorafur, prednisone, enalapril, and protonsil, which are converted to dopamine, oxazepam, phosphoramide mustard, ampicillin, fluorouracil, prednisolone, enalaprilat, and sulfanilamide, respectively [13]. CYPs, the most relevant drug-metabolizing enzymes, display inter-ethnic variability and inter-individual, due to CYP genes polymorphism and distribution of their common allelic variants in different populations [13]. The stress-mediated CYP genes regulation involves several mechanisms and is associated with activation of major hepatic signal transduction pathways, leading to the accumulation toxic metabolites, such as free radicals [13]. The oxidative stress, associated with increased release of cytokines/NF-κB signalling and thyroid hormones, insulin, and growth hormone modified secretion profiles have critical roles in CYP regulation. Although it has been demonstrated that stress alters the normal hepatic drug metabolism, stress is also associated with upregulation of the most CYP enzymes constitutive expression [15], such as that of CYP2D, which alternatively catalyses the brain synthesis of serotonin and dopamine [13], along with the hepatic and brain metabolism of the majority of antipsychotic, antidepressant, anxiolytic, and antiepileptic drugs [16]. The evaluation of the stress effect on drug metabolism has to consider that chronic uncontrolled stress is involved not only in the pathogenesis of metabolic syndrome but also in that of inflammatory diseases, depression, and cancer [17].

A relatively new notion is that of anaesthetic-induced developmental neurotoxicity (AIDN) correlated to surgeries performed in periods of time when the brain is highly vulnerable to environmental stress, such as young children, pregnant women, mainly related to the use of propofol (i.v.) and sevoflurane (volatile), with deleterious effects on neonatal brain development [18]. AIDN is characterized by cognitive sequelae due to enhanced neuronal apoptosis, decreased synaptogenesis and neurogenesis, along with neurodegenerative changes [18]. Recently, the key molecules involved in this disorder have been identified as non-coding RNAs (ncRNAs), which are not translated into proteins, being considered as tuners of cell fate. ncRNAs may represent possible markers for different type of brain disorders and also pharmacological targets for neuroprotective interventions or novel therapeutic approaches [18].

The benefits and side-effects of opioids are currently considered in the context of addiction and respiratory depression. A potentially lethal condition is that of opioid-induced respiratory depression (OIRD), mainly when the opioid is abused, in association with sedatives, alcohol, or illicit substances [19]. Recently, combination of opioids with respiratory stimulants has become a novel approach that enhances opioid safety, lowering OIRD occurrence and opioid death. However, considering the complex interaction between respiratory stimulants or reversal agents with the underlying opioid resulted in limited availability of such drugs. In order to understand these interactions, pharmacokinetic/pharmacodynamic (PKPD) modelling studies are performed, which may characterize the relationship between the reversal agent, respiratory depressant, and their effect, allowing their optimal manipulation in therapy.

Alcohol abuse is related to more pathologies than all other drugs combined, while alcohol dependence results in high rates of mortality and morbidity, being associated to adverse individual and social consequences. Alcohol use leads to alteration of the communication between brain neurotransmitters, hepatocytes alteration by alcohol’s by-products, allowing ammonia and manganese to cause hepatic encephalopathy, or increasing the risk of strokes, regardless of a history of coronary heart disease [20]. It is also correlated with foetal alcohol spectrum disorders and other birth defects, including foetal alcohol syndrome (FAS). Other pathologies related to alcohol consumption are that of fatty liver or liver steatosis, followed by alcoholic hepatitis, and fibrosis, resulting in cirrhosis, with a plethora of complications, such as insulin resistance, type 2 diabetes, jaundice, and liver cancer [20]. A serious condition associated to alcohol abuse is that of alcoholic cardiomyopathy that can lead to absolute heart failure or a disruption of the heart pacemaker, causing arrhythmias, such as atrial fibrillation and ventricular tachycardia, along with vessels elasticity loss, leading to hypertension, added to the release of stress hormones, leading to blood vessels constriction. Yet other conditions, such as pancreatitis, due to internal release of enzymes, added to acetaldehyde, is leading to pancreatic cells damage, with impact on the metabolic processes involving insulin. Last but not least, alcohol abuse leads to disturbances of both innate and adaptive immune systems. Thus, alcohol abuse can disrupt cytokines production, suppressing the T-cells development and NK cells’ ability to attack tumour cells. Consequently, cancer risk has also been attributed to alcohol abuse not only for liver but also for breast, oesophageal, laryngeal, oral, and pharyngeal cancer [21].

Nicotine, in association with oxidants, such as nitrogen oxides and free radicals, and carbon monoxide, contained by tobacco smoke (TS), is considered both neurotoxic and cardiotoxic [22]. TS has been associated with a high morbidity of traumatic brain injury, ischemic stroke, and various neurological diseases [23,24]. The vascular endothelial dysfunction is associated with nicotine exposure, ROS, oxidative stress-driven inflammation, and decreased activity of Na^+^/K^+^/2Cl^−^ cotransporter in the blood-brain barrier (BBB) [25]. Nicotine pharmacokinetics is correlated with its physicochemical properties and route of administration, with a rapid oral, intestinal, respiratory, and skin absorption, as well as the physiological system [26], with increased bioavailability via smoking or e-cig [27], reaching in seconds the arterial circulation and brain [24]. Its primarily metabolism is taking place in liver, leading to a major metabolite, cotinine, involving CYP450 2A6, aldehyde oxidase, flavin-containing monooxygenase 3, and uridine diphosphate-glucuronosyltransferase enzymes. Nicotine pharmacodynamics is based on its similar phenotype to that of endogenous neurotransmitter acetylcholine (Ach) and, consequently, its ability to bind to nicotinic acetylcholine receptors (nAchRs). Particularly, β2 containing nAchR mediates the reinforcing effects of nicotine. It has been demonstrated that nicotine long term exposure is correlated with strong desensitization of β2-type nAchRs, with a key role in nicotine addiction [26]. The reinforcement of nicotine effects is attributed to dopamine (DA) and dopaminergic pathways, while various factors have been demonstrated to affect nicotine metabolism, pharmacokinetics, and pharmacodynamics, such as physiological characteristics (gender, age, and diet), smoking behaviour, race, genetic polymorphism, medication, and certain diseases.

With a marked impact in therapy, recent knowledge in pharmacokinetics and pharmacodynamics has led to various strategies meant to counteract the low stability, low bioavailability, and poor solubility of oral anti-diabetics or to enhance the transdermal delivery of different active pharmaceutical ingredients by nanotechnology. Starting from the use of vesicular systems, such as conventional liposomes (firm lipid bilayered vesicles), the technology has been largely extended to niosomes (non-ionic surfactant distinct layer vesicles) [28] and to elastic liposomes, such as ethosomes and transferosomes (lipid-centered vesicular, pliable, and stress-receptive craters). Transferosomes can penetrate the skin as intact carriers of large proteins within 300 nm size up to high water content zones, such as the deep dermis and hypodermis [29].

Within this context, further research is needed for a deep insight into the complex influences of different types of stressors in pathology, along with the new tools of diagnosis currently available, and the possibilities to exploit their pharmacokinetics and pharmacodynamics, such as therapies using microparticles and nanoparticles. Furthermore, promising perspectives are emerging for early diagnosis, disease monitoring, and identification of complex pathways involved in their pathophysiology, along with histopathological and molecular characteristics in various stress-induced disorders, leading to novel, tailored therapeutic approaches.
